# Where Are We Now and Where Might We Be Headed in Understanding and Managing Brain Metastases in Colorectal Cancer Patients?

**DOI:** 10.1007/s11864-022-00982-0

**Published:** 2022-04-28

**Authors:** Ribal Bou Mjahed, Christoforos Astaras, Arnaud Roth, Thibaud Koessler

**Affiliations:** 1 Department of Oncology, University hospital of Geneva (HUG), Geneva, Switzerland; 2 Département de médecine interne - CHUV, Rue du Bugnon 21, CH-1011 Lausanne, Switzerland

**Keywords:** Colorectal cancer (CRC), Brain metastasis (BM), Metastatic pathways, Liquid biopsies, Novel treatments

## Abstract

Compared to liver and lung metastases, brain metastases (BMs) from colorectal cancer (CRC) are rare and remain poorly investigated despite the anticipated rise in their incidence. CRC patients bearing BM have a dismal prognosis with a median survival of 3–6 months, significantly lower than that of patients with BM from other primary tumors, and of those with metastatic CRC manifesting extracranially. While liver and lung metastases from CRC have more codified treatment strategies, there is no consensus regarding the treatment of BM in CRC, and their management follows the approaches of BM from other solid tumors. Therapeutic strategies are driven by the number and localisation of the lesion, consisting in local treatments such as surgery, stereotactic radiosurgery, or whole-brain radiotherapy. Novel treatment modalities are slowly finding their way into this shy unconsented armatorium including immunotherapy, monoclonal antibodies, tyrosine kinase inhibitors, or a combination of those, among others.

This article reviews the pioneering strategies aiming at understanding, diagnosing, and managing this disease, and discusses future directions, challenges, and potential innovations in each of these domains.

**Highlights:**

• With the increasing survival in CRC, brain and other rare/late-onset metastases are rising.

• Distal colon/rectal primary location, long-standing progressive lung metastases, and longer survival are risk factors for BM development in CRC.

• Late diagnosis and lack of consensus treatment strategies make BM-CRC diagnosis very dismal.

• Liquid biopsies using circulating tumor cells might offer excellent opportunities in the early diagnosis of BM-CRC and the search for therapeutic options.

• Multi-modality treatment including surgical metastatic resection, postoperative SRS with/without WBRT, and chemotherapy is the best current treatment option.

• Recent mid-sized clinical trials, case reports, and preclinical models show the potential of unconventional therapeutic approaches as monoclonal antibodies, targeted therapies, and immunotherapy.

**Figure:**
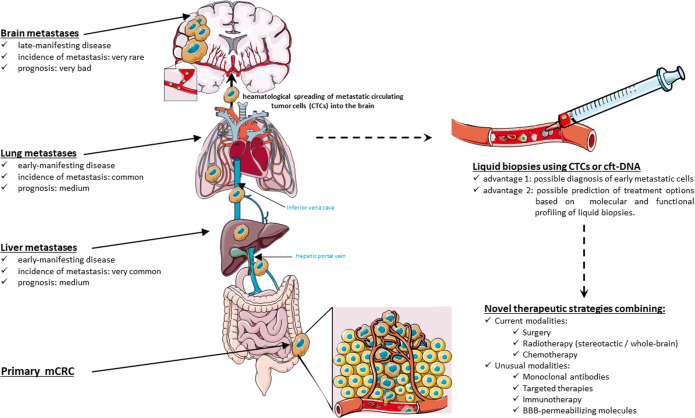
Graphical abstract

## Introduction

CRC is a major health concern worldwide, being the second and third most diagnosed cancer in women and men, respectively, and the fourth most common cancer-related cause of death [1].

While most CRC metastasize in the liver and/or the lungs, brain metastases (BMs) in CRC are very uncommon, especially when occurring without simultaneous extracranial disease [2]. Improvements in treatment options have altered the natural history of this disease by increasing survival, and with it the incidence of metastasis at previously uncommon sites, including the brain [3].

In this review, we firstly discuss the characteristics of CRC patients with BM, the risk factors for central nervous system (CNS) involvement, the characteristics of metastatic lesions, and prognostic factors. In the second part, we present the current pathophysiological hypotheses underlying the occurrence of BM and discuss the potential role of liquid biopsies in the early diagnosis of this disease. Finally, we investigate the molecular characteristics of the metastatic lesions and the potential targets for future pharmacological interventions.

## Patients and disease characteristics

BMs are the most common intracranial neoplasm in adults and are 10 times more frequent than primary tumors of the CNS, occurring in 20–40% of all cancer patients [4]. BM is a main feature of many primary cancers including lung (15–43%), breast (10%), testicles (15%), and melanoma (10%) [5]. CRC patients do not typically show BM until very late in the course of their disease [6]. The reported incidence ranges from 0.27 to 3% [2,3,7–11].

### Characteristics of the primary tumor

BMs are more commonly observed in patients with distal CRC compared to proximal ones, and primary tumors localized in the sigmoid and rectum account for 65–75% of the GI-metastatic lesions in the brain [2,10–14]. Most of the patients with BM had stage III (32–39%) or IV (39–56%) diseases [2,13–15], with grade 2 tumors (50–87%) [2,13–15]. Median age at the initial diagnosis of BM is below 60 years old (55–59 years old) [2,10,11,13–15], significantly lower than that of the CRC population [2,15]. Curiously, BMs occur more frequently in men than in women [2], although this observation might simply be the reflection of the distinct incidence of CRC in both genders.

### Risk factors

The absence of large-scale multi-centered prospective clinical studies as well as the scarcity of reports describing the host and tumor factors that might lead to the development of BM in CRC patients account for the current lack of risk factors. Left-sided tumors, long-standing pulmonary metastases (especially those with recent progression), and long survival are the current acknowledged risk factors [2,4,10,16–23]. Other associations with BM were confirmed by single groups on small samples and included the following: mutations in *PIK3CA* [17] and *BRAF* [18], overexpression of *EGFR* [19] and *CXCR4* [10], *MGMT* methylation [20], elevated CA19-9 [21] and CEA [22] levels. Most mCRC patients have elevated CEA, without significant difference between those harboring BM and those with exclusive extracranial progression [2]. Therefore, it was suggested that CEA should not be considered a specific marker for BM development but as a general indicator of tumor activity [2].

Curiously, Asian studies that included CRC patients reported lower BM incidence compared to North-American and European ones [2], although the role of ethnicity is still unclear in these regards.

### Characteristics of the metastatic disease

Compared to patients with non-GI metastatic tumors, mCRC patients have a smaller incidence of BM and longer intervals between primary diagnosis and BM development [9]. The majority (44–64%) of patients develop a single metastatic brain lesion [6,11–15], occurring mostly supratentorially (68.3–72%) [12,14,15]. On the other hand, cerebellar involvement is over-represented in BM from colorectal tumors, compared to BM from non-GI tumors [9,10,23].

Among mCRC patients bearing BM, only 1.75–10% do not show extracranial lesions [9,12,15,24–26]. Majority of patients with BM also present lungs (71–92%) [10,12–15] or liver (36.6–68%) metastases [12–15]. CRC patients with lung metastases might have higher risk of developing BM compared to those with uninvolved lung but metastatic liver. Studies have reported that the incidence of BM increased 2–10-fold when the lungs were involved [3,10,17,27]. Moreover, mCRC with BM show abundant incidence of lung (70–80%) but not liver (18–40%) metastasis at CRC diagnosis [2,10,14]. This is the opposite of the classical pattern of mCRC tropism consisting typically of liver metastasis in 70% of the cases while only 30% of the metastatic patients show lung involvement [28].

### Factors influencing BM-free intervals

BM-free interval (BMFI) is the time between CRC diagnosis and BM discovery. BMFI is significantly longer in patients bearing mCRC compared to non-GI tumors (35.40 months *versus* 6–8 months respectively) [9,12,13,15]. mCRC patients with longer BMFI showed higher OS [12,15]. The presence of extracranial metastases at the time of the CRC diagnosis shortened the BMFI significantly (8–9 months *versus* 40–56 months in metastases-free CRC at diagnosis), especially when occurring concomitantly in the lungs and the liver [12,15]. Primary tumor resection did not affect BMFI [12,15]. The role of adjuvant therapy remains controversial as recent studies suggest that both the administration of adjuvant therapy and the amount of chemotherapy given before the development of BM have less significance on the BMFI than previously expected. Indeed, it was shown that CRC patients who have received more than one line of chemotherapy have longer median BMFI compared to those receiving none or only a single line of chemotherapy. These differences were, however, not statistically significant (27–49 months depending on the treatment modality in treated patients *versus* 13 months in the untreated group) [12,14]. BMFI has increased dramatically over the last 30 years, thanks to the early detection of CRC as well as the improvement of treatment modalities [29].

These data should be handled with extreme care as these studies included few patients and are exclusively retrospective and were spread over several decades, during which and diagnostic tools have evolved radically.

### Prognostic factors for CRC patients with BM

BM from CRC are particularly aggressive. Among mCRC patients, those with BM have the lowest median survival (0.4–7.4 months versus 21–30 months) [2,4,11,14,24,30], even lower than that of patients with brain involvement from non-CRC primaries including the lungs, breast, and skin tumors (mOS: 9–12 months) [4,9,31]. Retrospective studies have shown that age, performance status, BM site, and BM number are prognostic factors associated to the OS [6,9,12,13,32]. Left-sided primary tumors shorten the overall survival by half with 6 months for right-sided CRC versus 3 months for the distal colon [12,13]. The presence of a metastatic disease at diagnosis significantly reduced the survival [12,13]. The increasing number of brain lesions significantly decreased the survival by up to two-thirds [9,12,13].

BM not amendable to targeted therapy decreased survival by over 80% [13]. PD-L1 positivity in the primary tumor was associated with lower survival [14], while *RAS* and *BRAF* status did not [14]. For the rare subpopulation of CRC with exclusive BM, the prognostic factors included age, performance status, and a controlled primary tumor [33].

## Biological mechanisms for BM development in CRC

While the understanding of the driving pathways for CNS involvement in CRC settings remains elusive, three different theories have been suggested and are presented here.

**Figure 1:**
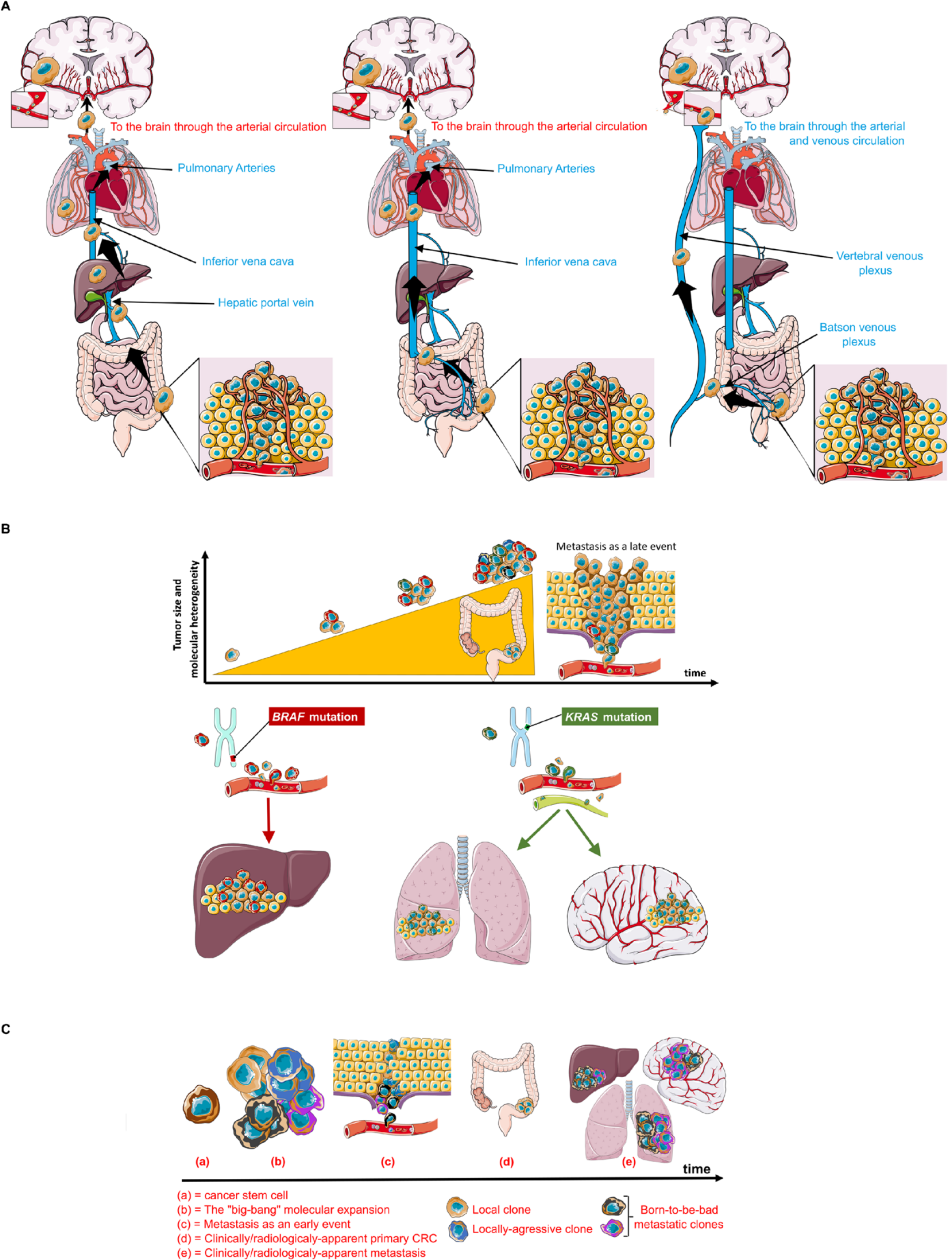
Anatomical, molecular, and tumor-specific features defining metastatic patterns observed in CRC. **A** Metastatic patterns in CRC as determined by the vascular theory. Metastatic cells spread hematologically towards the brain via different initial routes that will determine which other organs will be affected as spreading via the portal vein increases the risks for both liver and lung metastases (left panel), while its bypass spares the liver (middle panel). Tumor cells adopting the venous plexuses and spreading retrogradely in the supra-thoracic levels can reach the brain without involving both the liver and lungs (right panel). **B** Metastatic patterns in CRC as determined by the “seed and soil” theory. **C** Metastatic patterns in CRC as determined by the “big-bang” theory.

Firstly, the simplest yet most commonly accepted theory links CRC metastatic pattern to the **vascular anatomy** [6,15,34] (Fig. 1A). Indeed, CRC metastatic cells, known to spread haematologically, can adopt three different itineraries by passing through (i) the portal vein to the liver, then lungs and brain, respectively, which corresponds to the incidence and kinetics of metastatic findings in these three organs, or (ii) through the inferior vena cava then to lungs and brain, skipping the liver, or more rarely (iii) through a retrograde venous route *via* the vertebral plexus (i.e., Batson’s plexus) directly to the brain, bypassing extracranial organs. This theory could explain why patients with lung metastasis have a higher risk of developing BM than those with only liver involvement. It also explains why BMs are more frequent in left-sided CRC compared to right-sided ones, as the rectum drains directly into the inferior vena cava.

However, over one-third of brain metastases cannot be explained by the vascular theory, as different histological subtypes of a neoplasm in a given organ have different tropism characteristics; pulmonary tumors, for example, despite having equal access to the vasculature and thus equal chances for seeding the brain, show significant difference in BM incidence. Indeed, 40–% of SCLC patients develop BM while only 12–20% of NSCLC patients do [34]. This has led to establish the “seed-and-soil” theory (Fig. 1B) that links metastatic tropism to the molecular profile of the primary tumor and the metastatic cells. This hypothesis is supported by the fact that *RAS* mutations are more frequent in brain (and lung) metastases than in primary tumors [2,17]. In a study investigating the frequency of *RAS* and *PI3KCA* mutations and their influence on the metastatic pattern of CRC, *RAS mutations*, but not *PIK3CA*’s, correlated with brain (and lung) metastases, and were found more frequently in these lesions [17]. *BRAF* mutations on the other hand were associated with peritoneal involvement [18,35]. Surprisingly, *RAS* mutations are more frequent in right sided tumor which rarely metastasize to the brain [17].

While the “seed-and-soil” theory tells little about the timing of metastasis occurrence, it might rather assume that metastatic cells are genetically advanced cancer cells that evolved through a time-consuming series of sequential clonal expansion in the primary bed, before acquiring all the intrinsic mutations allowing them to proliferate in novel territories. This mechanism is thus supposed to happen while the tumor is already detectable or even advanced. However, preclinical models suggest otherwise as it has been shown that metastasis starts extremely early during the transformation process, and immunosurveillance keeps these occult metastatic tumors in an equilibrium state to limit their growth [36,37]. These discoveries challenge the seed-and-soil theory which cannot explain how a metastatic cell could build its entire mutational arsenal so early in the course of the disease.

Sottivera et al. suggested an alternative explanation in which tumors evolve in a “big-bang” fashion, rather than a linear one (Fig. 1C) [38]. The big-bang event (i.e., the transformation of the first cancer stem cells) results in an early tumor heterogeneity [38]. Among the evolving clones, some are destined for distant seeding and are considered the “born-to-be-bad” clones, responsible for early rather than late acquisition of invasive and metastatic potentials [38,39]. Along the same lines, recent work by Hu et al. showed that metastatic seeding of the brain (and the liver) in CRC patients occurs very early while the carcinoma is yet clinically undetectable, i.e., years before diagnosis and surgery [40^••^]. However, in their effort to identify biomarkers associated with metastasis, the authors identified molecular drivers for metastasis that appeared to greatly overlap with those of carcinoma initiation in mCRC, and were therefore not specific to metastasis [40^••^].

## BM are molecularly distinct from their primary tumors

The genomic landscape of CRC has been extensively studied and has identified several pharmacological targets. However, that of the brain lesions in CRC patients is not fully explored.

A recent sequencing study on metastatic samples from different organs in a small cohort of 17 mCRC patients concluded that the majority of the local and distant metastases in CRC arise from independent subclones within the primary tumor [41]. This suggests that the metastatic disease is heterogenous and exhibits genetic divergence in the course of its development, echoing the big-bang model discussed above [38, 39, 40^••^]. Whole-genome sequencing analysis of BM from various tumor types showed genetic distinction from their respective primaries as well as from their lymphatic and extracranial counterparts [42]. These data suggest that BM could be considered a distinct disease when it occurs.

In an effort to study the molecular profile of BM, Roussille et al. compared 38 paired BM and primary tumors and found different mutational frequency for *KRAS*, *NRAS*, and *BRAF* among these 2 groups [14]. *RAS* mutations were predominant and occurred more frequently in primary CRC with BM (62%) compared to those without BM (50%) [14]. Moreover, *RAS* mutations were significantly more frequent in BM compared to their paired primary tumors (85% *versus* 62% respectively) and 11% of the patients showed *KRAS* mutation in their BM despite a wild-type primary [14]. Other studies were in line with these observations despite the lack of paired analysis in some [43,44]. The authors also compared the tumor microenvironment characteristics in these two groups and showed that BM were two-fold less infiltrated by T-lymphocytes (CD3^+^ cells) and three times less infiltrated by cytotoxic T cells (CD8^+^ cells) when compared to their respective primary tumors (median rates; 15% *versus* 34% for CD3, and 3% *versus* 10% for CD8 infiltration respectively) [14]. These results do not suggest the brain to be an immune-desert, but rather reflect its subtle yet real different immune contexture [45,46]. Furthermore, 6% of tumors showed PD-L1 expression in the BM but not in the paired primary CRC [14].

Recent work by Sun et al. confirmed that brain lesions in CRC patients exhibit a diverse mutational pattern compared to their primary origin. In a cohort of 19 CRC patients with BM, they found elevated mutational signatures of homologous recombination deficiency (HRD) and mismatch repair deficiency (MMRd) in the brain biopsies but not in the respective primary tumors nor in the adjacent normal colon [47^•^]. Genomic analysis revealed BM-associated genes that carry frequent BM-specific mutations including SCNF family members 7A, 5A, and 2A (a voltage-gated sodium channel), as well as tumor suppressor genes *IKZF1* and *PDZRN4*, that are linked to metastasis in many cancers and associated with poor survival, especially when found in cerebral metastases [47^•^].

## The potential of liquid biopsies in the early diagnosis of CRC and the first metastatic cells

The conclusions drawn from the different driving pathways of metastasis in CRC as well as the molecular divergence between primary CRC and their respective BM might offer the rationale for using liquid biopsies in the early detection of CRC-derived metastatic cells as well as for molecular profiling of these cells. These biopsies detect and profile the following: circulating tumor cells (CTCs), tumor-derived cell-free products including tumor-associated DNA (cft-DNA), -miRNA, and -exosomes. Among all these biomarkers, experimental studies suggest CTCs and cft-DNA to have a great potential in metastatic CRC management.

### Circulating tumor cells

**Figure 2:**
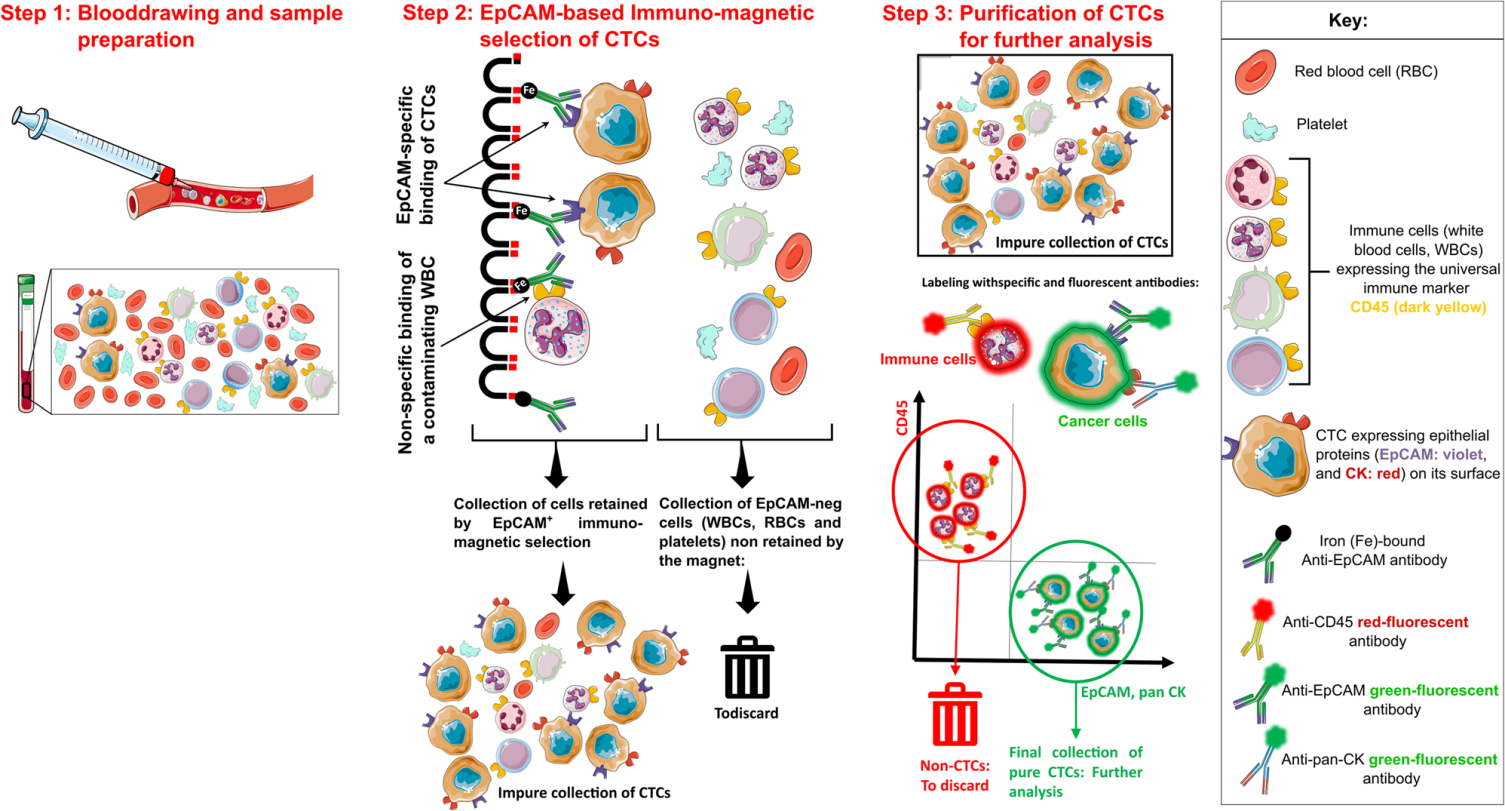
Extraction of circulating tumor cells (CTCs) as liquid biopsies using the CellSearch® system.

The extremely rare CTCs can be detected using immunomagnetic capturing techniques in which the antibodies recognize the Epithelial Cell Adhesion Marker (EpCAM) [48]. This has led to the development of the CellSearch® system (CS), the only FDA-approved method for CTC detection in CRC and other tumors to date [49]. EpCAM is an epithelial cell marker, rather than a cancer-specific one, but it is strongly expressed in most carcinomas including CRC. CS consists of utilizing EpCAM in a first step to detect carcinoma cells in the blood, followed by a “purification” step in which all contaminating non-tumor cells (mainly white blood cells (WBCs)) are eliminated (Fig. 2) [49,50].

CTCs can also be detected by PCR-based identification of genes expressed in epithelial cells or those associated with carcinoma, looking for specific DNA mutations, epigenetic modifications, or mRNA profiling. The most frequent parameter for detecting CTCs in CRC settings using this technique involves mRNA profiling of CK20 [51].

The Epispot assay is a third method for detecting the presence of viable CTCs by screening for the cancer-associated secretome in short-term *in vitro* cultures containing cells derived from the peripheral blood [52].

While most of these CTC-detecting techniques are essentially used as prognostic markers, very few investigated the potential in cancer screening, given the technological limits in both sensitivity and specificity. Tsai et al. described a very promising assay in which a small peripheral-blood volume was passed through a microfluidic chip coated with anti-EPCAM antibodies [53]. Using their assay as a screening test, they compared their results with colonoscopy and biopsy ones in 182 healthy controls, 111 participants with pre-cancerous lesions and 327 patients with CRC at different stages. They showed 88% of accuracy for all disease stages with very promising rates of false-positives and false-negatives. Overall, the sensitivity of their assay was 76.6% and specificity above 97% [53].

The key limitation of CTC-based technologies remains the low detection rate. This could be drawn back to (i) the rarity of these cells, as their estimated half-life is short in the bloodstream (1–2.4h) [54], and their frequency is small in various cancers including CRC (79–155 CTCs/mL) [55]; (ii) the strong contamination with WBCs (CTC:WBC lower than 1:10 [7]); (iii) the negative effect of first-pass in the liver, which would significantly decrease the number of CTCs in the peripheral *post*-hepatic circulation; and (iv) the reliance on EpCAM that might be lost when CTCs undergo a transition from epithelial to mesenchymal cells [56,57] or strongly downregulated upon entry into the blood stream [57].

To solve these shortcomings, different strategies have been tested: firstly, increasing blood volume to augment the detection rate. While this strategy augments the absolute number of CTCs, it does not affect the background noise caused by WBCs, which might explain the poor increase in detection rates observed when 30mL of blood was processed with the CS system, instead of the classical 7.5mL [58]. Cheng et al. described the Hydro-Seq technology using hydrodynamic scRNA-seq barcoding technique that enables accurate CTC separation in breast cancer patients without the contamination of WBCs and RBCs, allowing more specific transcriptome analysis in the isolated CTC population [59].

A second strategy to increase sensitivity is by drawing blood from the mesenteric vein that showed higher detection frequency and higher mean CTC counts when compared to blood drawn from the central venous circulation with the CS assay [60] or to peripheral blood using Epispot [52,61]. Contra intuitively, a meta-analysis of 36 studies with over 3000 CRC patients found that CTC detection in peripheral blood but not in mesenteric/portal blood correlated with poor prognosis [62]. Finally and in order to decrease EpCAM-derived bias, researchers have combined the CS assay with other EpCAM-independent technologies, which led to significant improvement in CTC detection [57,63].

The lack of commonly defined enrichment strategies and the low detection frequency remain the bottleneck for cell-based liquid biopsies. Future perspectives include the adoption of novel technologies tested in non-CRC patients [50] such as (i) leukapheresis [64] which can separate CTCs in large quantity and high quality [65] or (ii) functionalized catheters to capture CTCs in vivo using intravascular magnetic wires [66].

Finally, the substitution of the infrequent CTCs by tumor-derived endothelial cell clusters may present an alternative [67]. These benign cells are released from the tumor vasculature bed and show some phenotypic features of CTCs despite the absence of any evidence for cancer-related mutations. Experimental data allowed to trace these clusters back to their original tumor with high accuracy. The work by Cima et al. showed evidence of their potential use in early-stage tumors, as these clusters were significantly higher in stage I CRC patients compared to healthy volunteers [67].

The greatest potential for cellular-based liquid biopsies in mCRC lies in the direct analysis of CTCs both on the molecular level using genome- and transcriptome sequencing techniques, and on the functional one by investigating their proteome and differential secretome. Such profiling strategies allow to parse individual cells for clinically relevant molecular and functional information to allow better understanding of the initial disease, as well as better therapeutic predictions [68]. Indeed, isolated and enriched CTCs from CRC patients have been used to detect *KRAS* and *BRAF* mutations [69] showing 70% similitude in the *KRAS* mutation state compared to their primary tumors [69,70], but less consistent results in *BRAF* mutations [70].

To our knowledge, no study has investigated yet the use of CTCs in predicting BM in CRC.

While this application could be of great interest in detecting CTCs destined to the brain, several technical and theoretical challenges must be addressed first. One such concern includes the site from which blood should be drawn, as one could expect that the mesenteric vein could be enriched with CTCs directed to the liver, while blood from the vertebral plexus and past the pulmonary veins could be more interesting for BM prediction but more challenging to obtain. Moreover, would the discovery of CTCs past the pulmonary circulation correlate with the presence of BM? Do CTCs that are destined to the brain differ from those of the primary tumor? How about their resemblance to the actual brain bulk? Or to other CTCs destined to other organs? And finally, could these CTCs provide clues about the treatment options for BM eventually?

The divergent characteristics between cancer cells in the brain and the colon offer, theoretically, two great opportunities in the management of CRC patients with BM. The first is the early diagnosis as one can imagine that circulating tumor cells that present significantly higher *KRAS* mutations than those found in biopsies from the primary tumor could evidence higher risk for BM. Such finding should obviously be matched with the clinical symptoms of the patients, especially on the neurological level, for the elucidation of further diagnostic strategies. The second opportunity is the orientation of the therapeutic decision for these patients, as their BM could require different systemic treatments than those effective against the primary lesion.

### Circulating cell-free tumor-associated DNA

As with CTCs, cft-DNA is being extensively studied in mCRC settings as a minimal-invasive monitoring technique, and its different utilities will be discussed in this section.

Circulating DNA is not restricted to pathological conditions [71] but is mainly thought to be the result of an accelerated cell turnover like that observed in auto-inflammatory diseases [72] as well as cancer settings [73]. It consists of approximately 166 bp-long DNA fragments with an estimated half-life of 16 min to 2.5 h [74]. mCRC is believed to release the highest load of cft-DNA among the most common cancers [73], making this liquid biomarker of exclusive interest in mCRC as it reveals both its distinct genomic (mutations and chromosomal rearrangements) and epigenetic signatures. Indeed, Strickler et al. showed that the genomic landscape obtained by the molecular profiling of cft-DNA was highly concordant with tissue-based assays in treatment-naïve mCRC patients, despite the lack of detectability in the serum of 15% of these patients [75].

The abundance-quantification and qualitative genomic characterization of cft-DNA determine their monitoring potential:

i) Quantitatively speaking, the prognostic potential of cft-DNA has been addressed by many groups and has shown its ability to reflect on the presence of minimal residual disease [76] and predict recurrence in post-operative settings [76]. A relatively recent study investigating cft-DNA from 230 patients with stage II CRC has shown the capacity of liquid biopsies to outperform both radiology and CEA in detecting residual disease (50–85% specificity) and recurrence (cft-DNA: radiology lead time of 3–10 months) in both adjuvant chemotherapy and treatment-naïve groups, providing thus the opportunity for earlier introduction of a treatment or earlier change to the next line of therapy [77]. Moreover, studies have also shown the capacity of cft-DNA to determine the real-time efficacy of adjuvant treatments as their load will increase shortly after the administration of the cytolytic drug, and disappear in approximately 2 h, making them a key element in “quantifying” imminent treatment responses, offering critical information for early decision-making regarding the dosage as well as the continuation of a treatment [78–80].
ii) The qualitative approach for cft-DNA analysis consists of determining its mutational content to predict treatment responses and fine-tune the anti-cancer armatorium in a personalized fashion [81]. As cft-DNA molecules are believed to offer a better understanding of the molecular heterogeneity of the tumor, capturing the real-time tumor landscape and identifying “actionable-mutations” in cft-DNA is expected to provide better stratifications for patients that could benefit from more suitable and/or novel therapies. For example, patients harboring ERBB-2 amplifications have been successfully targeted with specific targeting agents in both the MyPathway [82] and HERACLES [83] trials. The latter has also shown that cft-DNA harboring this particular amplification was successfully detected in most of the patients, with a strong correlation between detection levels and treatment responses [83]. Similarly, a suitable application for cft-DNA concerns stratification for anti-EGFR (endothelial growth factor receptor) treatment, a widely used analysis that is almost exclusively conducted on solid biopsies, excluding thus patients with unreachable tumors, and discounting the molecular signature of the metastatic lesions. Given the reported 25% discordance for *KRAS* mutation status between primary and metastatic cancers [84], one could expect a significant number of patients with inaccessible brain metastases harboring wild-type *KRAS* but whose primary solid biopsies showed *KRAS* mutations, to be excluded from systemic EGFR treatment, disregarding hence the molecular characteristics of their metastatic lesions. Such features could have been predicted using the easily accessible cft-DNA from peripheral blood. More interestingly, cftDNA can also detect the changes in tumor heterogeneity due to clonal evolution during a specific treatment line: studies have shown that some patients acquire resistance to a first-line anti-EGFR treatment but undergo a re-sensitization after some subsequent EGFR-blockade-free lines, rendering them eligible, again, for anti-EGFR challenge [85]. These resistance-related heterogeneities can be hard to identify in tissue biopsies, especially in recurrent tumors, and can be detected using cft-DNA.

Despite the extensive use of cft-DNA in experimental studies, a major barrier impeding its routine clinical application includes low detection rates of cell-free DNA (cf-DNA) in general, and cft-DNA in particular. This is due to the fact that circulating DNA can be released from any cell type, healthy or not, mostly in extremely low quantities, and harboring low-frequency aberrations as well as non-neoplastic age-dependant alterations affecting common driver genes. Hence, improving the specificity and sensitivity remains a major goal that is currently fuelling the race to develop new “omic” approaches to address cft-DNA analysis for large-scale clinical purposes.

In terms of sensitivity, the classical gold standard qPCR approaches allowed cft-DNA investigations mainly in patients with advanced cancer stages harboring higher cft-DNA levels [86] but excluded patients with localized tumors that typically present cf-DNA:cft-DNA ratio lower than 0.001 and mutational alleles lower than 0.0001 [73,81]. New-generation PCR techniques such as digital PCR, among others [50], have overcome this hurdle and can detect mutant alleles with fractional abundance up to 0.00005 [50].

Concerning the specificity of cft-DNA analysis, their investigation is performed using targeted or untargeted approaches [87]; the former methods include PCR approaches and allow probing single or just few hot-spot mutations in *KRAS*, *BRAF*, or *EGFR* genes given their impact on the decision of therapeutic agents. The latter approaches include a variety on new techniques that allow a blinded landscaping of all the genetic aberrations present in the circulating DNA fragments, such as next-genome sequencing methods that can detect mutations, copy number alterations, translocations, transversions, inversion, and other chromosomal modifications [50]. Whole-exome and whole-genome sequencings offer an even broader spectrum of analysis as they can assess for aneuploidy and other chromosomal rearrangements, associated to tumorigenesis and present in either the initial clones or acquired as a resistance-related feature [50]. Finally, false positives can occur due to technique-based bias, age-related mutations, or chemo/radio-induced genetic alterations of normal somatic or germline cells. Some groups have suggested [88] and utilized [77] leukocyte DNA analysis to exclude false-positive mutations driven by clonal hematopoiesis.

In conclusion, the diagnostic, prognostic, and predictive impact of liquid biopsies with either CTCs or cft-DNA in mCRC keep being extensively studied despite an obvious lack in the direct comparison between these two sources. It could be safe to assume that cft-DNA could be closer to achieve clinical practice for mCRC due to their excellent suitability and higher sensitivity than CTCs, while the latter possess the unique potential for secretome/proteome/metabolome-based investigations in ex vivo culture and xenograft settings, allowing a better understanding of the real “behavior” of tumor cells, in the aim of developing more suitable therapeutic approaches.

## Treatment options for patients with BM from CRC

The treatment of mCRC has changed dramatically in the recent years, mainly with the rise of targeted therapies and immunotherapy. Treatment of CRC’s BM remains largely based on the molecular profile of the primary tumors.

While no consensus exists yet for those patients, their management follows the approaches of BM from other solid tumors and depends on factors including patient’s performance status, primary tumor’s characteristics, number/location of the brain lesions, and the presence of leptomeningeal disease [89]. Therapeutic objective is usually palliative and options encompass local strategies such as surgical resection, stereotactic radiosurgery (SRS), more organ-based treatment like whole-brain radiation therapy (WBRT), or their combinations [4,30,90]. Use of chemotherapy remains controversial due to its acknowledged limitation to effectively cross the blood-brain barrier (BBB) [4,13,30] and the fact that most of the patients have been already treated with multiple lines, and develop resistance.

A recent retrospective study presented at the ASCO meeting in 2020 investigated the treatment modalities of CRC patients with BM admitted at the Mayo clinic between 1994 and 2019 which included 104 patients. Multi-modality treatment including surgical brain lesion resection, postoperative SRS with/without WBRT, and chemotherapy significantly improved mOS (41 months for patients with multimodal treatment, 14 months for surgery and radiation, 12 months for chemotherapy and radiation, 5 months for surgery alone, 3 months for radiation alone, 0.4 months for best supportive care management) [11]. This study confirms that aggressive local treatment of BM using multi-modal approaches, when possible, could be beneficial and offer good clinical outcomes. The authors and others discuss that the need for aggressive local treatment is justified by the observations that local resections are associated with high rates of local recurrences in the BM in many cancer types [4,11,30,90].

Given the rarity of BM in CRC, there are no prospective controlled trials that have evaluated optimal treatment option and outcomes, outside of the local management strategies discussed above. Furthermore, randomized studies generally do not include patients with BM perpetuating our lack of knowledge [15,30].

New treatment modalities including immunotherapy, monoclonal antibodies, or tyrosine kinase inhibitors (TKI) have made their way into mCRC armatorium, without, once again, including patients harboring BM. The management of extracranial mCRC consists of a fluoropyrimidine (5-fluoruracil/capecitabine) combined to irinotecan or oxaliplatin and a monoclonal antibody (anti-VEGF or anti-EGFR), depending on (i) the localisation (right or left colon), (ii) the molecular profile (*KRAS*, *NRAS*, *BRAF* mutations), and (iii) treatment goal (maximum tumor shrinkage *versus* disease control) [91].

Next, we will discuss the cutting-edge treatment modalities as well as potential future ones to manage BM in mCRC patients.

### Monoclonal antibodies

Because of its high molecular weight, bevacizumab would be believed to be unable to penetrate the BBB and its use in CRC patients with BM was not considered for several years because of fear of risk of intra-cranial hemorrhages. A growing body of evidence suggests the efficacy of bevacizumab in BM from different primary tumors [92], including CRC. Chen et al. reported a shrinkage of the brain lesion in a CRC patient after using bevacizumab in combination with chemotherapy [93]. Similarly, Yoshida et al. reported to have successfully cured a patient with BM from rectal cancer by administrating bevacizumab and XELOX (capecitabine and oxaliplatin) [94]. Both studies claimed bevacizumab to be responsible of the intracerebral effect, although the mechanisms were not elucidated. The efficacy of these treatments might be due, at least in part, to BBB reconstitution and vascular normalization after disruption by metastatic cells. This is probably why the classical pharmacological concept of BBB penetration is not required for intra-tumoral diffusion in BM.

### Targeted therapy

In a cohort of 38 CRC patients bearing BM, HER-2 amplification was identified in the metastatic lesion of one of them (2.6%) [14], rendering anti-HER-2 a possible target when applicable. Patients harboring HER-2 amplification are usually treated using monoclonal antibodies directed against the extracellular domain of HER-2, such as trastuzumab or TKIs (lapatinib), targeting its intracellular catalytic kinase domain, hampering thus its signaling. Stemmler et al. measured trastuzumab levels in the serum and cerebrospinal fluid and found that it does not cross the BBB efficiently (ratio 420:1 respectively), unless combined with radiotherapy (ratio 76:1) [95]. On the other hand, TKIs (lapatinib) or the antibody-drug conjugate (trastuzumab-emtasine, T-DMI) seem to represent a viable option for the management of BM owing to their high BBB permeability as seen in preclinical models and phase II clinical trials in breast cancer patients [96]. Nevertheless, major question marks remain unanswered about their efficacy as solo agents.

Encorafenib is a TKI used in combination with cetuximab for the treatment of mCRC patients harboring a *BRAF*-V600E mutation. Its uptake across the BBB is estimated to reach 1–2% of its plasma concentration in mice, suggesting very poor penetration [97]. Nevertheless, a multicentred retrospective case series investigation of melanoma patients bearing that mutation and presenting BM who treated with a combination of encorafenib (BRAF-inhibitor) and binimetinib (MEK-inhibitor) reported 24% intracranial ORR, suggesting that the regimen is capable of penetrating the CNS [98].

In some CRC patients included in their cohort, Sun et al. identified BM-selective *BRCA1*/*BRCA2* mutations [47^•^], a homologous-recombinant deficiency that has been recently proposed as an ideal biomarker for NHEJ inhibitors like PARP inhibitors used in breast cancer [99]. However, most of the PARP inhibitors developed to date have a limited brain penetration due to the presence of P-glycoprotein (P-gp) and breast-cancer-resistance-protein (BCRP) on the BBB [100]. Different groups have reported novel PARP inhibitors with improved brain penetration in xenograft models in vivo as they are not substrates to P-gp nor BCRP, including AZD2461 or pamiparib, respectively [101,102]. These molecules have not yet been tested in BM from CRC.

### Immunotherapy

The observations that a subgroup of mCRC patients had BM infiltrated by cytotoxic T lymphocytes [14], expressed PD-L1 on tumor/stromal cells [14] or were enriched with MMRd-related genomic signatures that are associated to MSI [47^•^], suggest immunotherapy targeting the PD-1/PD-L1 axis as a potential therapeutic option. Immunotherapy using checkpoint inhibitors has always been considered to have limited potential in controlling brain metastases, but recent studies involving the tumor-microenvironment in BM showed that the brain is an immunologically distinct compartment rather than an immune-isolated one [103]. Indeed, immunotherapy using checkpoint blockade has shown intracranial activity in patients with secondary CNS lesions from clear-RCC, NSCLC, and melanoma [4,104^•^]. However, only limited data are available concerning immune-checkpoint blockade inhibitor in BM-CRC, and the few clinical studies available in mCRC show comparable efficacy of these biologic drugs on BM and other extra-cranial metastases [105].

### Blood-brain barrier permeability enhancers

One of the relatively recent approaches to increase BBB permeability to ensure better delivery of large-sized monoclonal antibodies into the CNS consists of transiently disrupting the BBB itself using hypertonic solutions. Mannitol for example draws water into the blood vessels from endothelial cells, causing them to shrink and allow the drugs through [106]. Mannitol is safe and well-tolerated in combination with systemic chemotherapy [106]. This approach showed encouraging effects using cetuximab [107] as well as bevacizumab [108] for the treatment of malignant glioma. BBB disruption also improved therapeutic effects of methotrexate and carboplatin on primary CNS lymphomas [109] and cerebral metastases [110].

## The increasing incidence of BM in CRC

Although poorly documented [2], many experts believe that BM incidence might be increasing over time, mainly due to the improved diagnosis of CRC at earlier stages, the intensive follow-up, and the resulting increased survival of patients [1,3,10]. In a study including CRC patients with rare metastatic sites, Sundermeyer et al. hypothesized that the early diagnosis and increasing numbers of available active systemic therapies for mCRC management improves survival and might therefore foster the development of previously occult sites of disease [3]. Interestingly, their data indicated a correlation between the incidence of bone metastases and the increased number irinotecan- or oxaliplatin-based cycles received, as these patients lived longer [3].

Other studies suggested that the true incidence of BM is likely underestimated, especially in retrospective studies collecting data before the 2000s, because tumor registries emphasize coding of the primary tumor over subsequent metastases [111]. Mackenzie et al. showed that 76% of CRC-BM patients were asymptomatic, and would not have been diagnosed with BM until the development of symptoms, suggesting subsequently a higher cumulative incidence of BM in the population of mCRC patients than historical reports would suggest [112].

Altogether, these data suggest that BM might be relatively early events in the evolution of metastatic CRC, with late clinical manifestation. Indeed, the symptoms indicating the involvement of rare metastatic sites in CRC might depend on the interactions between tumor cells and the remaining components of the tumor microenvironment including local specialized tissue, fibroblasts, blood/lymphatic vessels, and most importantly immune cells. This might explain how a microscopic lesion in the brain may be present at initial diagnosis and remain indolent for relatively long periods. Patients with similar scenarios may never develop clinically apparent disease in these sites. In such settings, one can imagine that improved therapy for the extracranial disease at its early stage would prolong the OS and may thus increase the incidence of clinically apparent BM.

## Conclusion

BM lesions from CRC are rare but are becoming more frequent with the increasing survival of mCRC patients. Compared to their primary tumors, BMs exhibit a diverse molecular pattern that could be potentially druggable. This emphasizes the need for molecular assessment techniques of the BM. New technologies such as CTCs or ctDNA technologies may have a huge impact on the stratification of CRC patients at risk of developing BM, as well as on the treatment options.

In conclusion, increasing the awareness that BM can occur in CRC patients, even silently, and the surveillance of those at risk of developing this rare metastatic profile could potentially lead to earlier detection and treatment. Patients with BM should be included in clinical trials to better define effective treatments and ultimately improve the prognosis of these patients.
